# Measuring disease-specific quality of life in rare populations: a practical approach to cross-cultural translation

**DOI:** 10.1186/1477-7525-7-92

**Published:** 2009-10-23

**Authors:** Victoria E Price, Robert J Klaassen, Paula HB Bolton-Maggs, John D Grainger, Christine Curtis, Cindy Wakefield, Gustavo Dufort, Arne Riedlinger, Christophe Soltner, Victor S Blanchette, Nancy L Young

**Affiliations:** 1 Division of Pediatric Hematology/Oncology, IWK Health Centre, Dalhousie University, Halifax, NS, Canada; 2 Division of Pediatric Hematology/Oncology, Children's Hospital of Eastern Ontario, Ottawa, ON, Canada; 3 Department of Clinical Hematology, Manchester Royal Infirmary, Manchester, UK; 4 Royal Manchester Children's Hospital, Manchester, UK; 5 Child Health Evaluative Science, The SickKids Research Institute, Toronto, ON, Canada; 6 Division of Pediatric Hematology/Oncology, The Hospital for Sick Children, Toronto, ON, Canada; 7 Pediatric Hemato-Oncology, Centro Hospitalario Pereira Rossell, Montevideo, Uruguay; 8 Department of Pediatrics, Charité - Universitätsmedizin, Berlin, Germany; 9 Anesthesiology and Surgical Intensive Care Department, University Hospital of Angers, Angers, France; 10 School of Rural and Northern Health, Laurentian University, Sudbury, ON, Canada

## Abstract

**Background:**

Disease-specific quality of life (QoL) measures have enhanced the capacity of outcome measures to evaluate subtle changes and differences between groups. However, when the specific disease is rare, the cohort of patients is small and international collaboration is often necessary to accomplish meaningful research. As many of the QoL measures have been developed in North American English, they require translation to ensure their usefulness in a multi-cultural and/or international society. Published guidelines provide formal methods to achieve cross-culturally comparable versions of a QoL tool. However, these guidelines describe a rigorous process that is not always feasible, particularly in rare disease groups. The objective of this manuscript is to describe the process that was developed to achieve accurate cross-cultural translations of a disease-specific QoL measure, to overcome the challenges of a small sample size, i.e. children with a rare disorder.

**Procedure:**

A measurement study was conducted in the United Kingdom (UK), France, Germany and Uruguay, during which the validated measure was translated into the languages of the respective countries.

**Results:**

This is a report of a modified, child-centric, cross-cultural translation and adaptation process in which culturally appropriate and methodologically valid translations of a disease-specific QoL measure, the Kids' ITP Tools (KIT), were performed in children with immune thrombocytopenic purpura (ITP). The KIT was translated from North American English into UK English, French, German, and Spanish.

**Conclusion:**

This study was a successful international collaboration. The modified process through which culturally appropriate and methodologically valid translations of QoL measures may be achieved in a pediatric population with a relatively rare disorder is reported.

## Introduction

The development of quality of life (QoL) measures has greatly enhanced the scope of outcomes that can now be measured by rigorous scientific methods. Their inclusion in clinical studies enables the assessment of differences and subtle changes between groups, either due to time or treatment, which was not previously possible. Changes in QoL are particularly salient to children and their families. As the cohort of patients with any rare disorder is small and even smaller when the focus is specifically children, international collaboration in the research of such disorders is essential as we work towards evidence-based practice. Consequently, it is recognised that QoL measures, many of which have been developed in North American English, require translation in order to retain their validity and reliability in a multi-cultural society and internationally.

In order to achieve accurate, cross-culturally comparable versions, formal methods of translation and adaptation need to be applied. This process involves both linguistic translation where the measure is literally translated into the new language and cultural translation where it is adjusted appropriately for the cultural context, as well as an evaluation process to ensure comparability with the original tool. Several publications have outlined processes for achieving this cross-cultural adaptation. Guillemin et al published a report in 1993, in which recommendations were proposed based on their review of the literature[1]. This was followed by two publications outlining guidelines for a comprehensive process of cross-cultural adaptation[2,3]. These guidelines and recommendations require the inclusion of large numbers of subjects across several countries and extensive use of professional translators. The recommendations from the published guidelines are summarized in Table 1 along with the modified process that is described in this report. Although these guidelines provide an excellent frame of reference, they are based on expert opinion. The best method to use is not clear. Furthermore, while these guidelines may be applicable when translating and adapting measures for large cohorts of adults, the process presents unique challenges when applied to children, and particularly when the measure is being developed for use in children with a rare disorder. Examples of such challenges include: 1) the difficulty in applying the guidelines to a small sample size (<50 subjects), and 2) the specific needs of children, including their unique linguistic style, which is difficult for professional translators to capture.

**Table 1:** A comparison of cross-cultural adaptations of quality of life measures.

	**Guillemin et al 1993 **[1]	**Bullinger et al 1998 **[3]	**Beaton et al 2002 **[2]	**Modified process for KIT**
**Background and methods**	Linguistic translation alone is insufficient for cross-cultural application of measures.	International Society for Quality of Life Assessment project group recommendations.	Based on Guillemin et al 1993.	Published guidelines too rigorous for a small cohort of children with a rare disease.
	Systematic literature review of the methodology of cross-cultural adaptation.	Focused primarily on the SF36, EORTC and Nottingham Health Profile experiences.	Guidelines Currently Used by the American Academy of Orthopedic Surgeons Outcomes Committee.	
	Proposed standardized guide-lines based on the review.			

**Forward translation**	≥ 2 Professional translators NSTL* and culturally representative.	≥ 2 Professional translators NSTL.	2 Professional translators (NSTL).	Single forward translation by bilingual clinical expert in target country.
	Both naive and informed translators are required.	Translators naïve to SF36.	Both naive and informed translators are required.	
	≥ 2 Independent translations.	Translators NSTL rate the difficulty of the translation.	2 Independent translations.	

**Consensus after forward translation**	No specific process outlined.	Within-country reconciliation of problematic items and response options with local PI and translators.	2 Translators and a facilitator synthesize a translated version of the questionnaire from the 2 forward translations.	Not part of the process.

**Back translation(final language into source language)**	≥ 2 Professional, naïve translators NSE.^#^	2 Professional translators NSE.	2 Professional, naïve translators NSE.	1 Professional, naïve translator for each of the target languages.
	Translate independently.	Health Assessment Lab compared back translations to initial version for "conceptual equivalence." Discrepancies discussed with local PI.	Translators should not have a clinical background.	Single back translation.
	Must have as many back translations as forward translations.		Must have as many back as forward translations.	

**Consensus/Synthesis**	A multi-disciplinary committee including, experts in concepts and the disease, to compare English versions.	International investigators meeting.	Expert committee consolidates all translations and produces a questionnaire for field-testing.	A multi-disciplinary expert committee compares English back-translated versions.
	Structured techniques are used to resolve discrepancies.		Expert committee consists of methodologists, health care professionals, language experts and translators.	Local bilingual clinical expert adjudicates any discrepancies with committee.
	Inclusion of bilingual members is ideal.			Consensus meeting to form reconciled versions.

**Testing with participants**	The translated questionnaire may be administered to a group of patients using a probe technique.	Focus groups with up to 50 respondents and translations revised as needed.	The translated version is administered to 30-40 people using a probe technique.	Training provided for cognitive debriefing skills by operations group.
	**or**	Testing done in individual countries.		Cognitive debriefing in individual countries.
	Both the English and translated questionnaires are administered to bilingual lay people.	Modified EORTC debriefing questionnaire used.		

**Total number of formal translations**	At least 2 forward and 2 back translations.	At least 2 forward translations and a consensus version, i.e. 3.	At least 2 forward translations and consensus version, i.e. 3.	Single forward and back translation.
		2 Back translations.	At least 2 back translations.	

**Number of consensus meetings**	After forward and back translations.	Between forward and back translations.	Between forward and back translations.	After forward and back translations.
	After pre-testing.	After difficulty ratings.	After back translations.	After pre-testing.
		After back translation.		

**Role of patients**	To ensure face validity of the translation.	Lay panel made up of general public was used to achieve consensus when translators could not.	To ensure translated version retains equivalence to original version.	To ensure face validity of the translation.
		Patients and lay people pilot tested final forward translation.		

**Number of patients**	Specific requirement is not Stipulated.	50	30-40	10 Children and their parents per country.

* NSTL = Native speakers of target language ^# ^NSE = Native speakers of English

To determine what was actually practiced in the field of measurement in the pediatric medical literature, we conducted a review of the literature from 1993 (when the first guidelines were published in the Journal of Clinical Epidemiology) to 2007. We identified 42 publications (birth to 12 years of age, 'validate' or 'validation' as a text word and Quality of Life exploded as a MESH term and cross-cultural comparison as a MESH term). Most of the publications were focused on generic measures, or measures that are applicable to large populations (e.g., asthma or arthritis). The fact that only one report was relevant to a rare condition, hemophilia,[4] underscores the barriers to cross-cultural translation in rare conditions.

The development of measures that can be used in multi-national studies is essential, particularly in rare disorders in children, since it is international collaboration that will enable new treatment interventions for rare disorders to be fully evaluated. Without cross-culturally validated measures, quality of life will be eliminated from such studies. However, the rigorous process described to date may prevent cross-cultural adaptations of questionnaires that are required in multilingual and multi-cultural research. Alternatively, some researchers may elect to implement QoL measures on which forward and backward translations have been performed, but that have not been tested with children, due to the constraints of a small sample size (for example, the Greek version of the PedsQL, ). Therefore, we propose a modified cross-cultural translation process for disease-specific pediatric QoL measures that is practical in the context of small sample sizes, yet is potentially capable of achieving the same high quality standards as have been previously published. The purpose of this report is to describe a process through which culturally appropriate and methodologically valid translations and adaptations of QoL measures may be achieved in a pediatric population with a relatively rare disorder, childhood immune thrombocytopenic purpura (ITP).

## Methods

**Figure 1:**
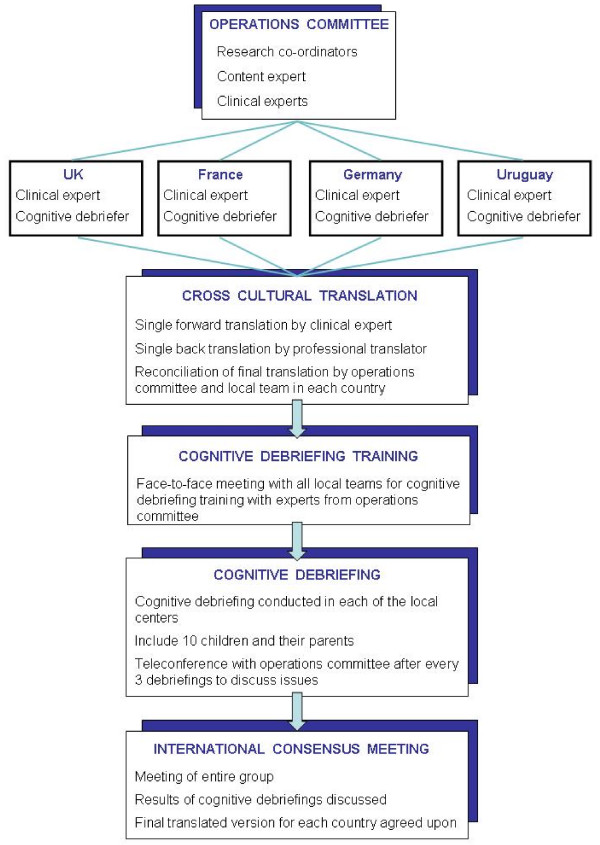
**The cross-cultural translation process of the KIT**.

We developed a modified process for cross-cultural translation and adaptation that factored into account the challenges of small sample size in rare disorders, and applied it to a quality of life tool. We used the KIT (Kids' ITP Tools), a disease-specific health-related QoL measure developed for children with ITP, originally developed and validated in North American English, as the example. This tool consists of three related measures: a child self-report, a parent report of their child's quality of life (proxy), and a parent-impact report[5]. The KIT was cross-culturally translated and adapted for use in the United Kingdom (UK), France, Germany, and Uruguay. Our approach followed previously published principles [2] and included five steps: 1) forward translation, 2) backward translation, 3) review of source and final translated version, 4) pre-testing for equivalence in source and final documents in the format of cognitive debriefing, and 5) an international consensus meeting. This process is presented in Figure 1.

### Step 1: Forward translation

A bilingual clinical expert, defined as a health care professional with clinical expertise in childhood ITP, in each of the target countries (UK, France, Germany, Uruguay) and who was also a native speaker of the target language translated the source document (North American-English KIT) into the target language, i.e. UK English, French, German or Spanish.

### Step 2: Backward Translation

A qualified professional translator (affiliated with a commercial translating service in Canada) performed a single backward translation into the original language, for each of the three target languages excluding UK English. The commercial translating service in Canada retains the services of international translators which ensured that the translations were completed in their natural milieu. The translators were also naïve to the objectives of the translation.

### Step 3: Review of source and translated version

A committee from the Canadian co-ordinating centre was formed to oversee the cross-cultural translation process at all four sites. The committee consisted of experts in the disease (ITP) as well as experts in the intent and content of the measure and the measure's development. A member of the committee (CC) compared the source document to each of the back translations and highlighted any differences. A bilingual health care professional with clinical expertise in the management of childhood ITP (an academic pediatric hematologist), whose mother tongue was the official language of the target country and who was not involved in steps 1 and 2, assisted the committee by adjudicating any discrepancies. The final adjudication process was conducted via teleconference with the clinical expert who had performed the forward translation in each of the target countries, the bilingual clinical expert from Canada and the study operations team. Consensus was met to create a reconciled version of the translated document.

### Step 4: Pre-testing

To ensure equivalence in linguistic, cultural and clinical context, cognitive debriefing was conducted in each of the countries following the methods described by Jobe[6]. The clinical experts from each of the countries identified a person who could perform the cognitive debriefings. The debriefers were fluent in their local language and dialect as well as fluent in English (written and spoken). The Canadian operations team met with the site leaders and cognitive debriefers from all of the countries, to deliver formal training and conduct practice training sessions in cognitive debriefing. Each of the 4 cognitive debriefers were expected to debrief at least 10 children (7 to 18 years of age), and 10 parents (some of whom represented children less than 7 years of age) in their home country. The children, who were invited to participate by each center, had either acute or chronic ITP and were willing to take part in the cognitive debriefing phase of the process of cross-cultural translation.

During each debriefing, the debriefer recorded whether or not each of the items was reported to be problematic in terms of the comprehension of the concept, the wording of the question, or the response options. They also recorded the response selected and any suggestions for improvements made by the respondents (e.g. more appropriate vocabulary). After at least three debriefings were completed, the local Canadian committee met via teleconference with the local clinical expert, and the cognitive debriefer in each of the target countries. The data collected were reviewed, any concerns that arose during the debriefing process were discussed and potential solutions were recommended. After consensus was reached, the agreed changes were implemented and used in the ensuing cognitive debriefings. This process was repeated until no new information emerged. The data collected by each of the cognitive debriefers were analysed by the Canadian co-ordinating team to identify items in which >10% of children or >10% of parents in any country reported difficulties. We postulated that the cognitive debriefing of 10 children and 10 parents would be sufficient for the pre-testing phase of the cross-cultural translation process.

### Step 5: International Consensus Group Meeting

Finally, the entire group (Canadian operations team as well as the teams from UK, Germany and Uruguay) met at an international consensus meeting. The items that were identified as needing revision during the cognitive debriefing process in each of the four countries were discussed along with potential solutions, and collective decisions were made. These decisions established the final version of the measure for each of the target countries.

## Results

The process of forward translation, backwards translation and cognitive debriefing was conducted for the three related measures (child version, parent-proxy version and parent-impact version) in four countries over 10 months. In total, eight clinical experts (four from the Canadian operations team and one from each of the target countries), three professional translators (one each for French, German and Spanish) and four cognitive debriefers (one each from UK, France, Germany and Uruguay), 39 children with ITP and 67 parent-child pairs participated. The mean age of the complete sample of children was 10.2 years (range 5-15 years). Of the 39 children who provided data (i.e. completed the child version), the mean age was 11.9 years (range 7-17 years) and the male-female ratio was 1:1.9. Twelve children with acute ITP and 27 children with chronic ITP were included.

Table 2 demonstrates the proportion of respondents identifying areas of concern in the newly translated KIT. Overall, 69% of children identified some areas for improvement in the child version of the KIT, 39% of parents identified some areas of question in the proxy version of the KIT, and 69% of parents identified some questions to be changed in the parent-QoL measure. Changes were suggested for 10 of the 26 KIT items: seven were identified only by children, one was identified only by parents and two were identified by both children and parent proxies. It became apparent that the problematic items were consistent between the child and proxy version and 8 of the 10 problems were identified in two or more countries. Changes were also suggested for 9 of the 26 parent-KIT items; eight of these were identified by parents in two or more countries. Problems encountered in achieving cross-cultural equivalence included discrepancies in semantic equivalence (vocabulary and grammar), idiomatic equivalence (idioms and colloquialisms) and conceptual equivalence (disease-related). An example of a difference in semantics was the statement "I felt sick," referring to feeling "unwell," which was interpreted by UK patients, as "I felt nauseated." An example of a substitution to achieve idiomatic equivalence was the word "cranky," a North American colloquialism that was translated into "bad tempered/moody" in UK English, "bizarre" in French, "genervt" in German and "irritable" in Spanish. An example of conceptual equivalence was found in the concept of receiving "treatment through an IV" and "taking medicine by mouth," which was foreign to UK, French and German patients, as they are not commonly treated with medication when thrombocytopenic. Furthermore, "staying overnight in hospital" was the norm for patients interviewed in Uruguay, but this was not a common practice for patients in the UK, France and Germany.

**Table 2:** Proportion of respondents identifying problems in the KIT

**Country**	**Children**		**Proxy**		**Parents**	
France	60%	(9/15)	29%	(6/21)	54%	(13/24)

Germany	80%	(4/5)	50%	(6/12)	100%	(12/12)

UK	100%	(7/7)	73%	(8/11)	64%	(7/11)

Uruguay	58%	(7/12)	25%	(5/20)	70%	(14/20)

**TOTAL**	**69%**	**(27/39)**	**39%**	**(25/64)**	**69%**	**(46/67)**

Common semantic issues also arose with respect to the response options. The response "seldom" translated differently in each country and was often back translated to "rarely." For example, when "seldom" was translated into French it became 'rarement,' in Spanish it became "raremente," and in German it became "selten". All three of these words were back translated to "rarely." The UK group identified concerns with the comprehension of "seldom" after their third cognitive debriefing and elected to change "seldom" to "rarely" in the UK version.

After an international consensus meeting, consensus was met to institute changes to the questionnaires to achieve improved linguistic and cultural translations. The response option "seldom" was changed to "rarely" in all translations, except the German and North American-English questionnaires, as the cognitive debriefing had confirmed that "seldom" was understood by the children and parents in these countries.

Three changes were made to the parent-impact version: 1) the word "bother" was changed to "worry" in all of the translations, 2) the phrase "did the uncertainty about" was changed to "did not knowing about" in the UK version, and 3) "note, you may provide other comments about your child's ITP at the end of the questionnaire" was added to all versions. The unit of measurement in which a platelet count is reported was changed to reflect each country's standard. In the UK-English version "IV" was changed to "drip."

## Discussion

We developed a modified process for the cross-cultural translation of a quality of life tool that takes into account the challenges of small sample size in a relatively rare disorder, namely ITP, occurring during childhood. The method we developed is a simplified form of the rigorous process for translation of measures, outlined in previously published guidelines [1-3].

A single forward and back translation was performed for each of the target languages in contrast to the guidelines that suggest at least two of each translation is performed. Bilingual clinical experts from each of the countries (pediatric academic hematologists) completed the forward translations, while professional translators were used for the back translations. Our method required a local academic pediatric hematologist, fluent in the source and target languages, to participate in a teleconference to facilitate the reconciliation process. We found the role of the clinical experts to be essential to the process for several reasons: they are pediatricians who by definition are experts in child development and therefore helpful in achieving age-appropriate translations, as well as understanding the specialized terminology used in disease-specific measures. The clinician in the target country, together with his/her counterpart in Canada, further assisted the process by identifying linguistically correct translations that needed to be modified to achieve accurate, culturally acceptable translation, e.g. "klinik" vs "poliklinik" in German. This process identified that not only were accurate child-centric linguistic and cultural translations required, but in fact a medical/clinical translation was also necessary. An example of this can be found in the short form "IV," which is a North American abbreviation for "intravenous catheter." The equivalent word for IV is "drip" in the UK, and there was not a direct translation for this in French. The inclusion of the clinical experts in our translation process provided the necessary detail, and replaced the need for multiple forward and back translations and a second commercial translator.

The cognitive debriefing process identified important linguistic and cultural issues, many of which were specific to children. We believe that these nuances would have been challenging for a professional commercial translator to identify and this again underscores the role of the bilingual clinical experts as part of the Canadian operations team. Most of the issues were resolved by regular teleconferences between the operations team and the investigators in the target country. The local bilingual clinical experts participated in relevant meetings/teleconferences to assist with translation, ensure accuracy and avoid any misunderstandings. Changes were made after consensus was met regarding an issue and were incorporated in the ensuing debriefings. This process provided continued surveillance of the linguistic/cultural/medical translation. Any remaining issues were resolved at an international consensus meeting. The result was four versions of the KIT (comprised of the child, parent-proxy and parent-impact versions) with linguistically, culturally and clinically appropriate modifications.

Through this process we identified a third aspect of translation. Translation into the new clinical context was essential, in addition to the linguistic and cultural translations. The management of childhood ITP differs from country to country, therefore the process had to include the appropriate translation of terminology to accommodate the different approaches to the management of childhood ITP. An example of this is that the majority of children diagnosed with ITP in the UK would be observed only, whereas many of these children in North America would receive active treatment. Although the translated KITs have not been validated yet, we believe that despite the different treatment approaches, the same QoL tool will be used and understood by all patients.

There is no evidence that any one of the previously published guidelines is superior and as suggested by Perneger et al "more empirical research is needed to understand what translation methods yield the best results."[7] A simple linguistic translation may not provide an accurate translation process. We identified two key problems with the published guidelines during the cross-cultural translation of the KIT. First, our cohort of patients was smaller than the 30-50 patients suggested in the guidelines. As ITP is a rare disorder in childhood, it would be unrealistic to assume such a large cohort. Secondly, the specific linguistic styles of children may have been lost during the translation process by commercial translators. In this regard, we believe the roles of the bilingual clinical experts to be imperative. These experts ensured accurate linguistic and cultural translations and avoided the need for at least two forward and back translations as per the published guidelines. A modified linguistic translation with cultural adaptation may represent an achievable process, which is a compromise between a simple linguistic translation and the standard provided by the published guidelines. We believe that this modified, child-centric approach to the cross-cultural translation of the KIT will prove to be as accurate as the processes previously published. This successful international collaboration has resulted in the adapted process proposed here being used as a guide for similar studies. The common measure that resulted from this process has undergone validation and will be reported.

## Competing interests

The authors declare that they have no competing interests.

## Authors' contributions

VEP: acquisition and analysis of data, drafting of manuscript. RJK: conception and design, acquisition of data, analysis and interpretation of data, drafting the manuscript, and critical revision of manuscript for important intellectual content. PHB B-M: acquisition of data, critical revision of manuscript for important intellectual content. JDG: acquisition and interpretation of data, critical revision of manuscript for important intellectual content. CC: acquisition and analysis of data, critical revision of manuscript for important intellectual content. CW: acquisition and analysis of data, critical revision of manuscript for important intellectual content. GD: acquisition of data, critical revision of manuscript for important intellectual content. AR: acquisition of data, critical revision of manuscript for important intellectual content. CS: acquisition of data, critical revision of manuscript for important intellectual content. VSB: interpretation of data, drafting the manuscript and critical revision of manuscript for important intellectual content. NLY: conception and design, acquisition of data, analysis and interpretation of data, drafting the manuscript, and critical revision of manuscript for important intellectual content. All authors have given their final approval of this version of the manuscript to be published.
